# Hierarchical porous carbon foam electrodes fabricated from waste polyurethane elastomer template for electric double-layer capacitors

**DOI:** 10.1038/s41598-022-16006-8

**Published:** 2022-07-11

**Authors:** Mahitha Udayakumar, Pál Tóth, Henrik Wiinikka, Jaskaran Singh Malhotra, Blaz Likozar, Saso Gyergyek, Anett Katalin Leskó, Ravikumar Thangaraj, Zoltán Németh

**Affiliations:** 1 Advanced Materials and Intelligent Technologies Higher Education and Industrial Cooperation Centre, University of Miskolc, Miskolc, 3515 Hungary; 2 Institute of Chemistry, University of Miskolc, Miskolc, 3515 Hungary; 3 Institute of Physical Metallurgy, Metal Forming and Nanotechnology, University of Miskolc, Miskolc, 3515 Hungary; 4 RISE Energy Technology Center, Box 726, 941 28 Piteå, Sweden; 5 Division of Energy Science, Department of Engineering Sciences and Mathematics, Luleå University of Technology, 97187 Luleå, Sweden; 6 DTU Offshore, Technical University of Denmark, Elektrovej, Building 375, 2800 Kongens Lyngby, Denmark; 7 Department of Catalysis and Chemical Reaction Engineering, National Institute of Chemistry, Hajdrihova 19, 1001 Ljubljana, Slovenia; 8 Institute of Energy and Quality Affairs, University of Miskolc, Miskolc, 3515 Hungary

**Keywords:** Materials for energy and catalysis, Energy, Environmental chemistry

## Abstract

Plastic waste has become a major global environmental concern. The utilization of solid waste-derived porous carbon for energy storage has received widespread attention in recent times. Herein, we report the comparison of electrochemical performance of porous carbon foams (CFs) produced from waste polyurethane (PU) elastomer templates via two different activation pathways. Electric double-layer capacitors (EDLCs) fabricated from the carbon foam exhibited a gravimetric capacitance of 74.4 F/g at 0.1 A/g. High packing density due to the presence of carbon spheres in the hierarchical structure offered excellent volumetric capacitance of 134.7 F/cm^3^ at 0.1 A/g. Besides, the CF-based EDLCs exhibited Coulombic efficiency close to 100% and showed stable cyclic performance for 5000 charge–discharge cycles with good capacitance retention of 97.7% at 3 A/g. Low equivalent series resistance (1.05 Ω) and charge transfer resistance (0.23 Ω) due to the extensive presence of hydroxyl functional groups contributed to attaining high power (48.89 kW/kg). Based on the preferred properties such as high specific surface area, hierarchical pore structure, surface functionalities, low metallic impurities, high conductivity and desirable capacitive behaviour, the CF prepared from waste PU elastomers have shown potential to be adopted as electrodes in EDLCs.

## Introduction

Supercapacitors (SCs), also known as ultracapacitors are advanced energy storage devices that can be charged-discharged in seconds and have potential in power-demanding applications such as heavy-duty electric vehicles, electromechanical devices, non-intermittent electricity from renewable sources, etc.^1,2^. Based on the charge storage mechanisms and device characteristics, supercapacitors can be classified into three categories: (i) electric double-layer capacitors (EDLCs), (ii) pseudocapacitors, and (iii) asymmetric capacitors^3–6^. Although pseudocapacitors have high specific capacitance and store energy mainly through Faradaic charge transfer between the electrode and electrolyte^7^, they have limitations in practical applications due to poor cycle stability and high cost. Carbon-based EDLCs continue to dominate the commercial market due to fast pulses of energy, long cycle life and high Coulombic efficiency^8^. Here, we focus on symmetric EDLCs that store and release energy via the physical adsorption–desorption of ions on the surface, forming an electric double layer at the electrode–electrolyte interface^9^. Porous carbons stand out as promising electrode materials for EDLCs because of their high specific surface area, good electrical conductivity, physicochemical stability, ease of preparation and low cost^10^. Carbon-based electrode materials are not only excellent candidates for EDLCs but also play an important role in supporting the active material of pseudocapacitors. Most commercial supercapacitors use biomass-based activated carbons derived from coconut shells, wood, bamboo and sawdust as electrodes^11^ – these suffer from low specific capacitance and poor rate capability.

Besides high specific surface area, pore size and pore geometry influence the electric double layer. The specific capacitance of EDLCs is mainly determined by the effective specific surface area and pore size distribution (micro-, meso- and macropores) of the porous carbon electrodes. Through the increased surface area, micropores improve electrochemical performance; however, micropores may limit the diffusion and transport of ions – carbon materials that only contain micropores often fail to meet the requirements for high-performance supercapacitors^12^. Nevertheless, hierarchical structure in form of meso- and macropores interconnected with micropores shorten the diffusion path and may facilitate ion transport^13^. Thus, amorphous carbons with a hierarchical pore structure comprising well-developed networks of pores and channels are highly suitable for EDLCs.

The conversion of waste materials into porous carbon electrodes for EDLCs is an important area of research. Recently, various biomass and agricultural wastes have been used to produce hierarchical porous carbons (HPCs) e.g., waste coffee grounds^14^, sword bean shells^15^, soybean dreg^16^, corn husk^17^, corn cob and leaf ^18^, wheat straw^18^, foxtail grasses^19^, mangosteen peel^20^, mango stone^21^, Lentinus edodes ^22^, etc. Besides, Wu. et al.^23^ converted waste sugar solution into HPCs for EDLCs and investigated their electrochemical performances in aqueous and ionic electrolytes. Ma et al.^24^ used a one-pot green synthesis strategy to produce nitrogen-doped HPC using a recyclable salt template and applied it as electrodes in a symmetric supercapacitor. Apart from biomass wastes, hazardous oily sludge waste^25^, solid leather waste^26^ and non-biodegradable plastic wastes^27^ were used as precursors for producing HPCs for supercapacitors. Plastic waste has become a major global environmental concern. Approximately 380 million tons of plastic waste are produced annually, of which at least 10 million tons end up in the ocean^28^. To effectively recycle them, many researchers have focused on the transformation of plastic wastes into valuable carbon materials and their applications as electrodes for energy storage devices. Hierarchical porous carbon nanosheets were produced from ‘real world’ mixed plastic wastes using organically-modified montmorillonite catalytic templates and applied as electrodes for supercapacitors^29^. Moreover, HPC-based electrodes derived from the most common plastic wastes such as polyethylene terephthalate (PET)^30,31^, polyethylene^32,33^, polypropylene^34^, polystyrene^35,36^, polyacrylonitrile^37^, polyurethane (PU)^38^ and melamine foams^12^ were reported.

Polyurethanes (PUs) are ubiquitous in many applications. In 2019, approximately 8% of the PU produced in Europe, the Middle East and Africa were elastomers^39^. PU elastomers are high-performance materials with a wide range of applications as films, elastic fibres, medical tubes and shoe soles^40^. They also find applications in other industrial sectors such as the construction, aerospace or automotive industry due to their large elastic deformation and excellent noise and vibration damping capabilities. Their extensive usage results in the generation of significant amounts of PU elastomer scraps that are not easily degradable.

In this work, the electrochemical performance of CFs produced from waste PU elastomers using a previously reported technique^41^ was evaluated in an aqueous KOH electrolyte to explore potential utilization in EDLCs. The applicability of this type of waste polymer as an energy storage material was investigated in line with multiple Sustainable Development Goals of the United Nations, e.g., Goals 7 (“Affordable and Clean Energy”) and 12 (“Responsible Consumption and Production”)^42^. As such, the presented research simultaneously targets the objectives of energy and the circular economy.

## Methods

### Materials

Waste PU elastomers (C-59.5%, H-7.91%, N-6.26%, S- < 1.0%, O-25.3%, data from CHNS-O analyzer) collected from Elastico Ltd., conc. sulfuric acid – 96 wt.% (VWR International Ltd.), sucrose (Magyar Cukor Zrt.), nitrogen and carbon dioxide cylinders (purity > 99.995%) (Messer Group GmbH.) were used. Carbon black (CB), polyvinylidene difluoride (PVDF), N-methyl-2-pyrrolidone (NMP) and potassium hydroxide (KOH) were purchased from Alfa Aesar. All chemicals were used as received without any further purification. CR2032 coin cell cases were procured from PI-KEM.

### Fabrication of CF-based symmetric two-electrode coin cells

#### Synthesis of CFs from waste PU elastomer

 The CFs were prepared using the waste PU elastomer template following two different activation approaches, the one-step & the two-step processes, as reported in our earlier study ^41^. A brief description is given below:

- Foam pretreatment – The PU elastomer was cut into cubes and immersed in acidified sucrose solution (2.5 g/mL) for 12 h followed by room drying overnight and cured in a hot air oven at 110 °C for 10 h.
- One-step process (named ‘EFAC1’) – The treated foams were directly activated in CO_2_ (flow rate—200 mL/min) at 1000 °C (heating rate – 10 °C/min) for 100 min.
- Two-step process (named ‘EFAC2’) – The treated foams were first pyrolyzed in N_2_ at 900 °C (heating rate – 10 °C/min) for 60 min followed by activation in CO_2_ (flowrate—200 mL/min) at 1000 °C (heating rate – 10 °C/min) for 100 min.

#### Coin-cell fabrication

**Figure 1:**
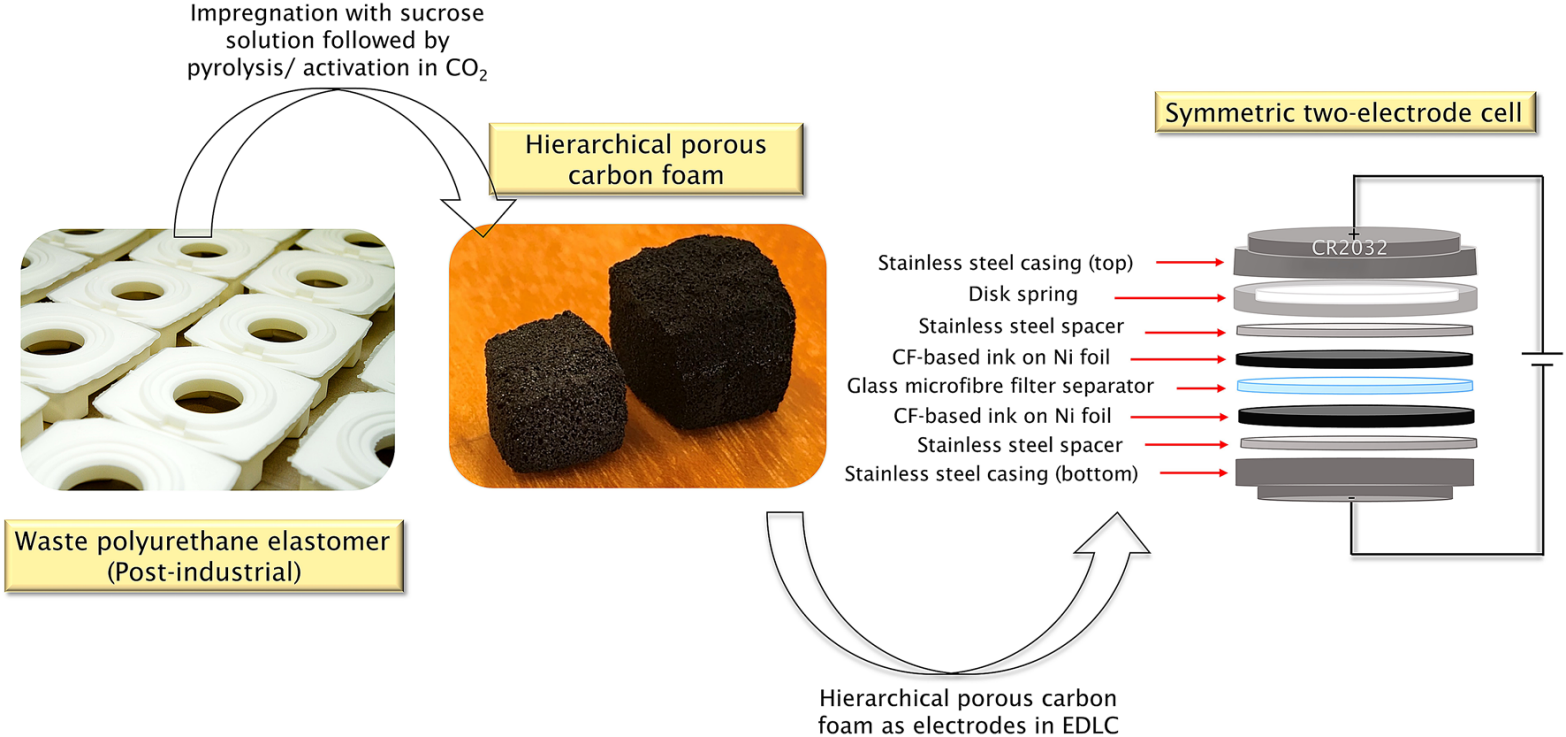
Schematic representation of waste polyurethane elastomer-based CF electrode for a symmetric two-electrode cell.

 Symmetric two-electrode cells were fabricated to analyse the charge-storage capabilities of electrodes based on EFAC1 and EFAC2 in an aqueous, 6 M KOH electrolyte. The electrode inks were prepared by mixing the CFs (70%) with PVDF (20%) and Carbon Black (10%) with a total weight of 0.4 g in 4 mL NMP. PVDF acts as a binder, while Carbon Black is a conductivity enhancement agent. The homogeneous ink was cast on Ni foil (current collector) using a doctor blade setup and dried in an oven. The average thickness of the films was 40 μm. Circular electrodes of 12 mm diameter were obtained using a steel punch. The effective mass of active material on each electrode was ~ 2.4 mg. Two electrodes with very similar active material masses were chosen and pressed at 6.9 MPa. Before assembly, 14 mm glass microfibre filter (Whatman) separators were soaked in a 6 M KOH electrolyte. CR2032 coin cell cases were used for the fabrication of supercapacitors. The assembled cells were pressed at 5.2 MPa using a crimper and the cells were left for 24 h to allow the electrodes to sufficiently soak the electrolyte before measurement. The schematic representation of waste polyurethane elastomer-based CF electrode assembled in a symmetric two-electrode cell is shown in Fig. 1.

### Characterization of physical and chemical properties

The surface morphology of the porous CFs was investigated by Transmission Electron Microscope (Jeol JEM-2010F). The samples were prepared by dispersing the CF powders in isopropanol, dropping the suspension on the Cu-supported amorphous C film and drying the suspension under ambient conditions. The pore size distribution of CFs was determined using the ImageJ software^43^ from TEM micrographs. N_2_ and CO_2_ adsorption–desorption experiments were carried out at 77 K and 273 K, respectively to determine the specific surface area (SSA) (ASAP 2020, Micromeritics Instrument Corp. USA). Before each measurement, the samples were degassed by holding at 90 °C for 24 h. Micropore volume was determined from the application of the t-plot method and Dubinin-Astakhov equation to the adsorption isotherm of N_2_ at 77 K and CO_2_ at 273 K, respectively. The structural properties of the CFs were determined by the powder X-ray diffraction method (XRD) using a Bruker D8 Advance diffractometer with a Cu K-α radiation source (40 kV and 40 mA) in parallel beam geometry (Göbel mirror) with a position-sensitive detector (Vantec1, 1° opening). Measurements were taken in the 2–100° 2θ range with a goniometer speed of 0.007° 2θ/14 s. Samples were top-loaded on zero background Si sample holders. Raman spectroscopy measurements were carried out using a high-resolution Raman spectrometer (Nicolet Almega XR, Thermo Electron Corporation, Waltham, MA, USA) equipped with a 532 nm Nd:YAG laser (50 mW). The X-ray photoelectron spectroscopy (XPS) analyses were carried out by the PHI-TFA XPS spectrometer (Physical Electronics Inc) equipped with an Al-monochromatic source. The analysed area was 0.4 mm in diameter and the analysed depth was about 3–5 nm. The high-energy resolution spectra were acquired with an energy analyzer operating at a resolution of about 0.6 eV and pass energy of 29 eV. The accuracy of binding energies was about ± 0.3 eV. To study surface chemistry, high-energy resolution XPS spectra C 1 s, O 1 s and N 1 s were measured and decomposed into different peaks related to different bonding of elements on the surface. The wettability test was carried out using the sessile drop method (SP 12 melt microscope—Sunplant Ltd., Hungary), via the acquisition of a silhouette shot. This method measures the angle of the sessile drop resting on the flat surface of the CF (polished using emery cloth sheets) using a goniometer – a microscope equipped with a video camera and a suitable magnifying lens, interfaced with a computer running image analysis software (KSV Instrument Ltd., Finland) was used to determine the tangent angle. Quantitative determination of metallic impurities of the CFs was conducted using a Varian 720 ES inductively coupled plasma optical emission spectrometer (ICP-OES) using the Merck Certipur ICP multi-element standard IV. For ICP-OES analysis, a pre-treatment method of dry ashing coupled with acid extraction was used: 2 g samples were placed in a crucible and ashed in a muffle furnace at 900 °C for 3 h. After cooling, the crucibles were washed with 6 mL of 2.0 wt% diluted nitric acid and heated on a hot plate for 20 min at 110 °C. The extracted solutions were diluted up to 25 mL with ultrapure water and then analysed by ICP-OES.

### Characterization of electrochemical properties

The electrochemical performance was evaluated using symmetric two-electrode systems and 6 M KOH electrolyte via cyclic voltammetry (CV), galvanostatic charge–discharge (GCD), and electrochemical impedance spectroscopy (EIS) using a Biologic potentiostat workstation. CV was carried out at different scan rates (5 to 200 mV/s) in the potential window of 0 to 0.8 V. The scan rate (mV/s) was kept constant for each measurement. Charge–discharge measurements were performed at different current densities from 0.1 to 10 A/g within the potential limits of 0 to 1 V.

The gravimetric specific capacitance (C_sp_g_, F/g) and volumetric specific capacitance (C_sp_v_, F/cm^3^) of the CF-based electrodes were calculated from each charge–discharge curve using Eqs. (1), and (2) respectively:

1 $$ C_{sp\_g} = \frac{2 \times I \times \Delta t}{{m \times \Delta V}} ,$$

2 $$ C_{sp\_v} = C_{sp\_g} * \rho, $$

where I is the constant discharge current (A), Δt is the discharge time (s), m is the mass of active material on a single electrode (kg) and ΔV is the potential window of the discharge voltage (V). The CF density ρ was reported in our earlier study ^41^ (EFAC1 – 1.81 g/cm^3^ and EFAC2 – 1.78 g/cm^3^).

The Coulombic efficiency (η) is defined as the ratio of discharging time to the charging time when the charge–discharge current densities are equal ^44^ and the parameter was calculated using Eq. (3).

3 $$ \eta = \frac{{t_{D} }}{{t_{C} }} \times 100\%, $$

where t_D_ and t_C_ are the discharge and charge times (s). The Coulombic efficiency is usually used for evaluating the cycle stability of electrode materials and devices by comparing the first and the last cycle.

The energy density (E, Wh/kg) of the device was calculated using Eqs. (4), and (5).

4 $$ E = \frac{1}{2} \times \frac{{C \times \Delta V^{2} }}{m \times 3600} , $$

5 $$ C = \frac{I \times \Delta t}{{\Delta V}} ,$$

where C is the capacitance of the device (F). m is the total mass of the active material in both electrodes (kg).

The power density (P, W/kg) is defined as,

6 $$ P = \frac{E}{\Delta t} . $$

Cyclability and capacity retention of the supercapacitor was analysed by carrying out charge–discharge measurements over 5000 cycles at 3 A/g.

Electrochemical impedance analysis was performed in the frequency range of 100 kHz to 0.1 Hz with an AC signal of 10 mV amplitude. The maximum power (P_max_, W/kg) of the device was calculated as

7 $$ P_{\max } = \frac{{\Delta V^{2} }}{4 \times m \times ESR} , $$

where ESR (Ω) is the equivalence series resistance obtained from the impedance spectrum and m is the total mass of the active material in both electrodes (kg).

## Results and discussion

For a better understanding of the correlation between structure and function, the physicochemical properties of the carbon foams were widely studied and the results are discussed here:

### Morphology, textural and surface properties of CFs

**Figure 2:**
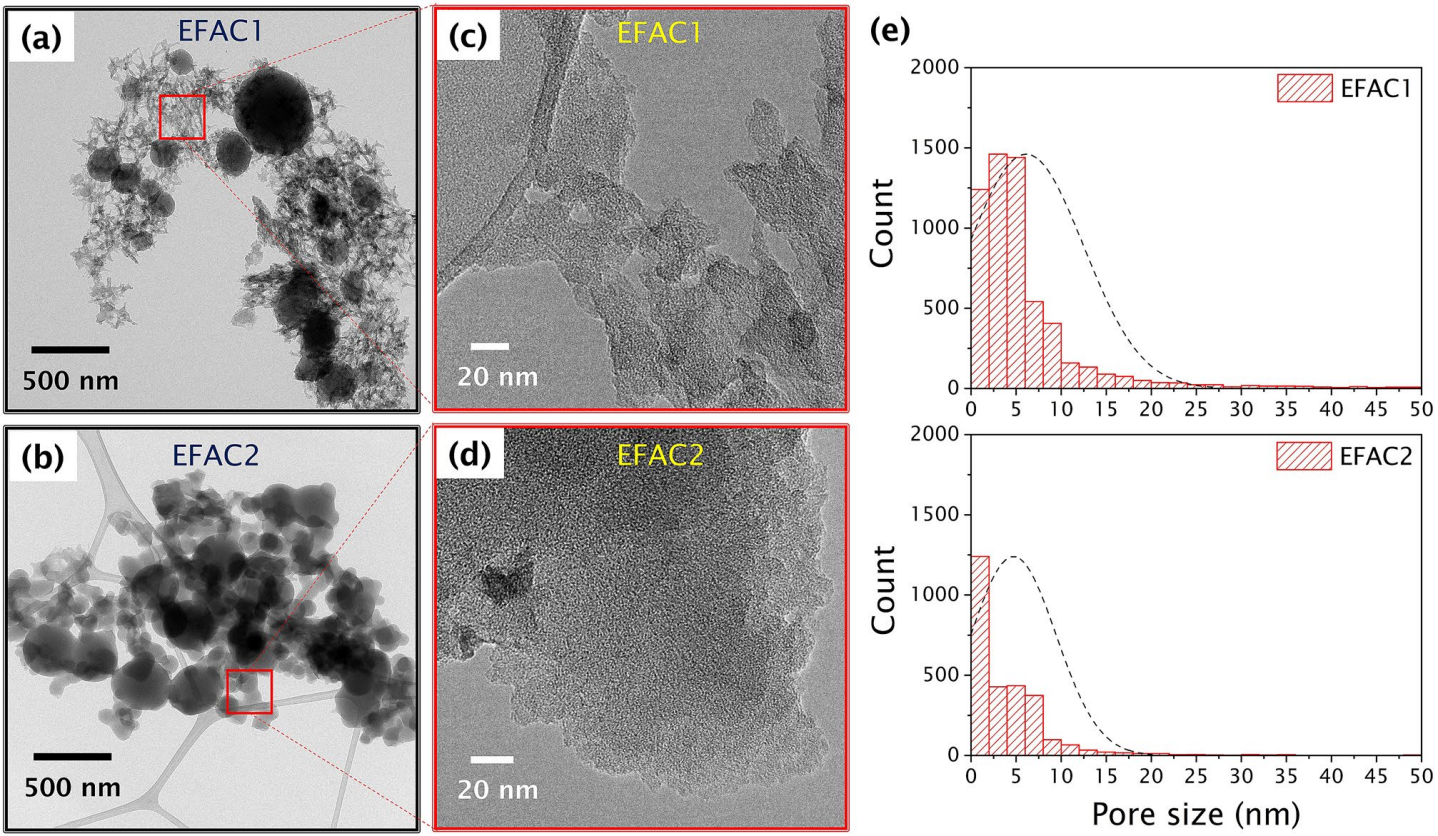
**(a,b)** Transmission electron microscope (TEM) images, **(c,d)** High resolution (HRTEM) images, and **(e)** pore size distribution of the porous CFs.

The TEM and high-resolution micrographs of the porous CFs are shown in Fig. 2. EFAC1 has an interconnected fibrous and porous structure consisting of numerous visible mesopores and macropores whereas EFAC2 is less porous and contains denser carbon particles. The high magnified images of both samples (Fig. 2c,d) show the amorphous nature of carbon sheets with micropores and less graphitic carbon on the edges. The pore size distribution of EFAC1 in Fig. 2e reveals the presence of well-distributed micro-mesopores than EFAC2. Moreover, these carbonaceous materials are composed of numerous nano-sized carbon particles of varying sizes, irregularly shaped sheets and thin irregularly shaped particles. Figure 2a has shown the higher porosity of EFAC1, however, the spherical carbon particles seem dense and non-porous. Both materials possess an amorphous or turbostratic structure and their structural properties analysed by XRD and Raman are provided in supplementary Fig. S1a and b online, respectively.

The specific surface area and micropore volume of CFs calculated from the adsorbed volume of N_2_ at 77 K and CO_2_ at 273 K are given in Table 1. Micropore volumes (V_m_ (N_2_) and V_m_ (CO_2_)) were determined from the application of the t-plot method and Dubinin-Astakhov equation to the adsorption isotherm of N_2_ at 77 K and CO_2_ at 273 K, respectively. V_m_ (N_2_) represents the total volume of micropores including the supermicropores (0.7 – 2 nm), whereas V_m_ (CO_2_) obtained from the very low relative pressure range (P/P_0_ = 0 – 0.03) covered at 273 K (the respective isotherm is given in supplementary Fig. S2 online), corresponds to the volume of narrow micropores (up to 0.7 nm). The remaining pore volume of V_0.97_ – V_m_ (N_2_) represents the volume of mesopores and macropores.

**Table 1:** Textural properties of CFs.

Sample	N_2_ (77 K)			CO_2_ (273 K)	
	S_tot_ (N_2_) (m^2^/g)	V_m_ (N_2_) (cm^3^/g)	V_0.97_*−V_m_ (N_2_) (cm^3^/g)	S_tot_CO2_ (CO_2_) (m^2^/g)	V_m_ (CO_2_) (cm^3^/g)
EFAC1	2068	0.52	0.56	1042	0.42
EFAC2	1263	0.36	0.11	1247	0.5

* Total pore volume at P/P_0_ ~ 0.97.

From the sorption data of N_2_ and CO_2_, the following interpretations were made:

*Sample EFAC1* The SSA and V_m_ calculated from N_2_ (77 K) > CO_2_ (273 K), as the direct activation in CO_2_ creates wider micropores and mesopores. The N_2_ can also fill the wider micropores whereas CO_2_ fills only narrow micropores or is adsorbed by a surface coverage mechanism^45^, which shows the difference in values measured with the two adsorptive.

*Sample EFAC2* The SSA calculated from the adsorbed volume of N_2_ at 77 K and CO_2_ at 273 K is consistent for EFAC2, but the V_m_ of EFAC2 measured by N_2_ was lower than CO_2_ due to the restricted diffusion of N_2_ in narrow micropores at 77 K.

Therefore, both the adsorptive provide a better knowledge of the whole porosity range of the CFs. EFAC1 has wider and heterogeneous micro-and mesopores with a lower volume of narrow porosity, whereas EFAC2 has a higher volume of narrow microporosity and lower volume of wider micropores with a very limited meso-macropores. The porosity and the surface area of EFAC1 were higher than EFAC2 and the reason behind such behaviour was reported in our earlier study^41^. These two parameters are directly related to capacitance, as the charge stored on the electrode surface depends on the contact between the electrode and electrolyte^46^.

**Figure 3:**
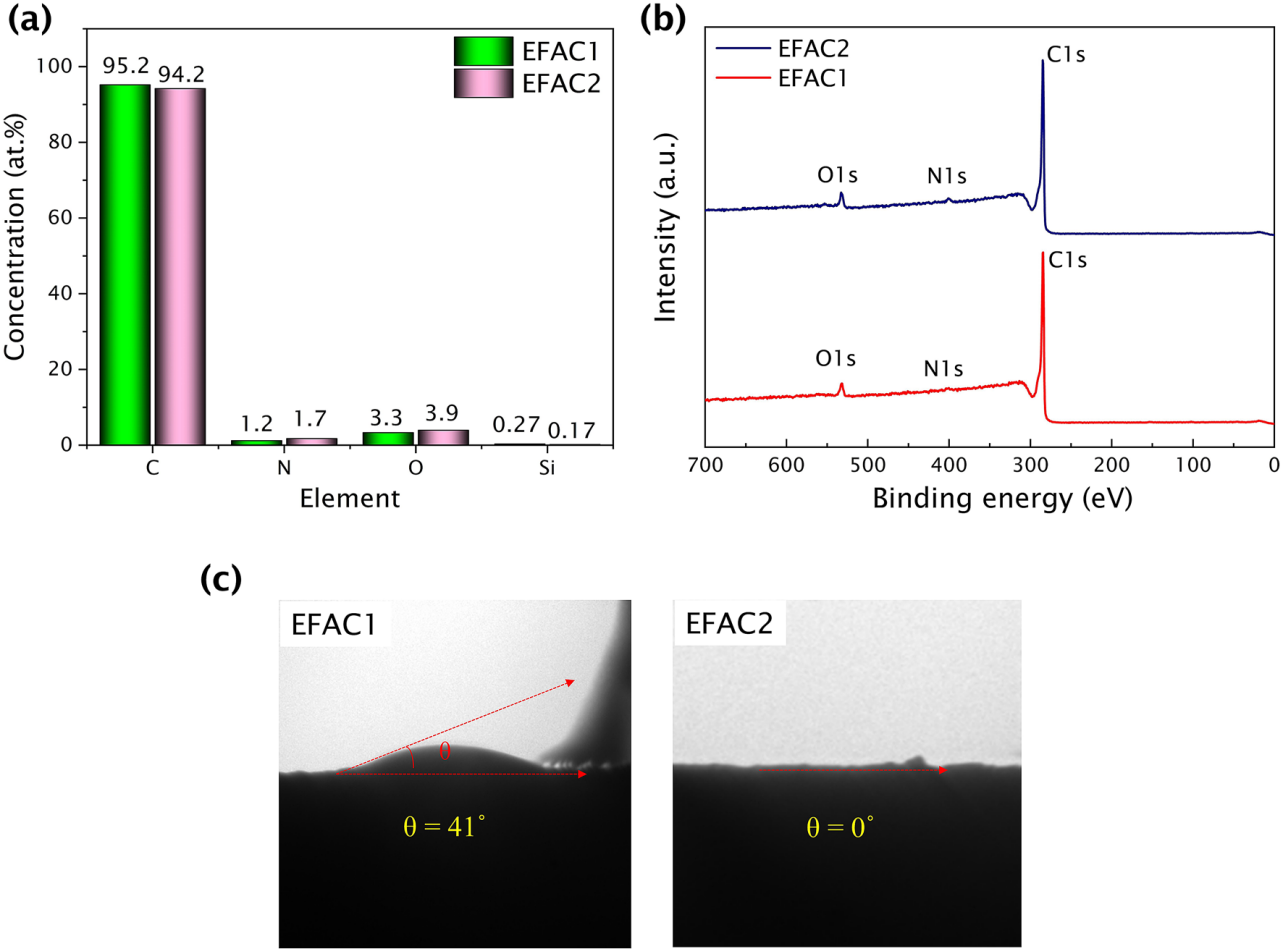
**(a)** XPS surface composition, **(b)** survey spectra, and **(c)** contact angle of the CFs.

On the other hand, the surface properties also influence the electrochemical performance of the carbon electrodes. Therefore, the surface chemistry of the CFs was investigated by XPS. The surface elemental composition and the survey spectra are shown in Fig. 3a and b, respectively.

The relative concentration of functional groups is given in Table 2. A detailed discussion of the XPS results and the deconvoluted peaks (C 1 s, O 1 s, and N 1 s) are given in supplementary Fig. S3 online. Based on the peaks at 286.5 eV and 532.7 eV, it seems the relative concentration of the hydroxyl functional group of EFAC2 is higher than EFAC1, which enhanced the wettability of EFAC2 (θ = 0°) ^41^ as shown by the contact angle measurement in Fig. 3c.

**Table 2:** Deconvoluted data of C 1s, O 1s and N 1s peaks.

Peaks		Bonds	EFAC1 (%)	EFAC2 (%)
C 1s	284.4 eV	C = C	54.5	54.7
	285.3 eV	C–C/ C–H	18.5	18.8
	286.5 eV	C–OH	10.4	11.0
	287.9 eV	C = O/O–C–O	5.8	5.7
	289.3 eV	O = C–O	5.4	4.9
	291 eV	π–π*	5.4	4.8
O 1s	530.8 eV	O = C/O–C–O	34.3	20.1
	532.7 eV	C–OH	55.5	73.6
	535.4 eV	C–O–C	10.2	6.3
N 1s	398.5 eV	pyridinic N	27	30
	400.9 eV	graphitic N	73	70

Carbon purity is a key factor for EDLCs with long cycle life and it is recommended to minimize the heavy metal content in all manufacturing steps of electrodes ^47^. Hence, the metal ion impurities of the CFs were analysed using the ICP-OES technique and the results are given in supplementary Fig. S4 online. The metallic impurities of CFs were between 15–110 ppm, which is 25–40 times smaller than the metallic impurities (Ca, Si, Mg, Fe, Al, Mn, Ba, Sr) of commercial biomass-derived AC ^48^.

### Electrochemical performance of CFs

To investigate the charge-storage properties of the as-prepared porous CFs, symmetric two-electrode cells were fabricated. The assembled two-electrode cells were analysed by cyclic voltammetry, galvanostatic charge–discharge cycling and electrochemical impedance spectroscopy.

**Figure 4:**
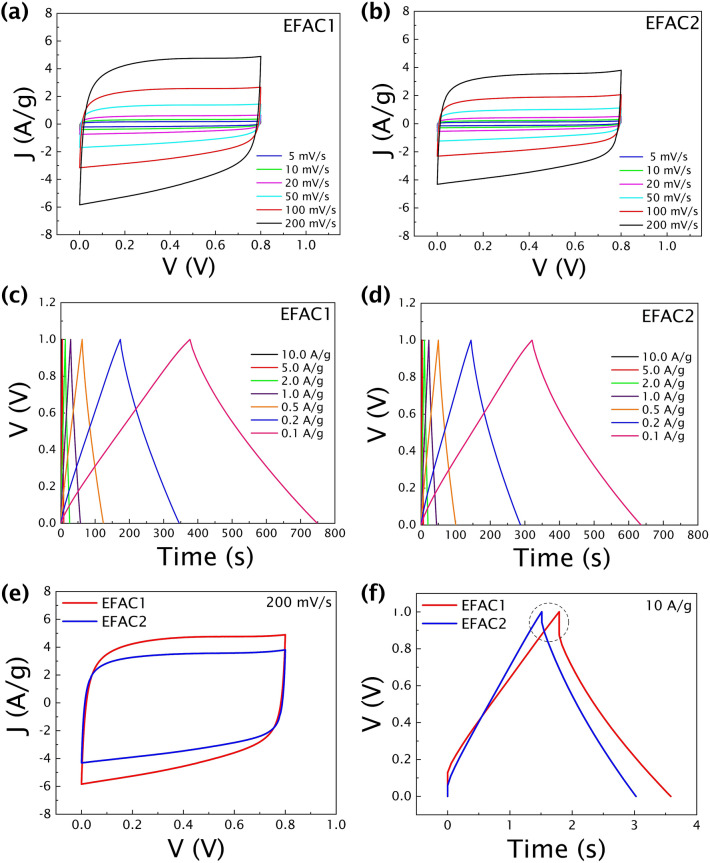
**(a,b)** Cyclic voltammograms at different scan rates, and **(c,d)** charge–discharge profiles at different current densities of the symmetric two-electrode cells, **(e)** comparison of the two materials in CV curves at a high scan rate of 200 mV/s, and **(f)** charge–discharge profile at 10 A/g showing IR drop.

Figures 4a and b show the cyclic voltammograms of EFAC1 and EFAC2 at different scan rates (5 to 200 mV/s) in the potential window of 0 to 0.8 V. The obtained cyclic voltammograms are symmetric and nearly rectangular at all scan rates. These rectangular shapes of the CV curves indicate ideal capacitive behaviour, revealing good charge transfer and showing the effective double-layer charge storage mechanism. In all cases, the capacitive current increases with the scan rates. In comparison with EFAC2, the EFAC1 has a larger area enclosed by CV and hence the highest capacitance at all scan rates. Though some Faradaic behaviour can be observed through the wide redox peaks on account of some redox processes of O- and N- heteroatoms present in the carbon framework, the primary contribution to capacitance comes from non-Faradaic EDLC behaviour due to enhanced surface area and electrostatic interaction of oxygen-containing functional groups with the aqueous electrolyte. As both materials have a higher percentage of the –OH group compared to other functional groups, the pseudo-capacitive behaviour might be because of the reaction > C–OH ↔  > C = O + H^+^  + e^-^ at the electrode interfaces ^49^.

Figures 4c and d show the galvanostatic charge–discharge curves of EFAC1 and EFAC2, respectively. The GCD curves exhibit good symmetry and nearly linear discharge slopes that confirms good electrochemical reversibility and Coulombic efficiency. Both electrode materials exhibit good stability at different current densities, as depicted by nearly triangular charge–discharge curves at all current densities. Thus, the GCD curves show the good rate capability of the supercapacitor at different current densities, which indicates good charge storage behaviour.

The performance of the two materials is compared by the CV (at a scan rate of 200 mV/s) and GCD (at a current density of 10 A/g) curves as shown in Fig. 4e and f. Even at a high scan rate of 200 mV/s and current density as high as 10 A/g, both materials show quasi-rectangular-shaped CV curves and nearly triangular-shaped GCD curves. Such behaviour of the double-layer capacitor suggests a high rate capability and low internal resistance. However, there is a little IR drop (Fig. 4f) during the changing of polarity, which is smaller for EFAC2 indicating the higher diffusion rate of electrolyte ions as it contains more –OH group than EFAC1. Due to the high electronegativity of the oxygen atom in the –OH group, the bond between the oxygen and hydrogen is highly polar, which acts as an H-bond acceptor and donor. Hence it strongly attracts solvated electrolyte ions, enhancing the wettability of the carbon in the aqueous electrolyte and thus faster ion transport in EFAC2.

**Figure 5:**
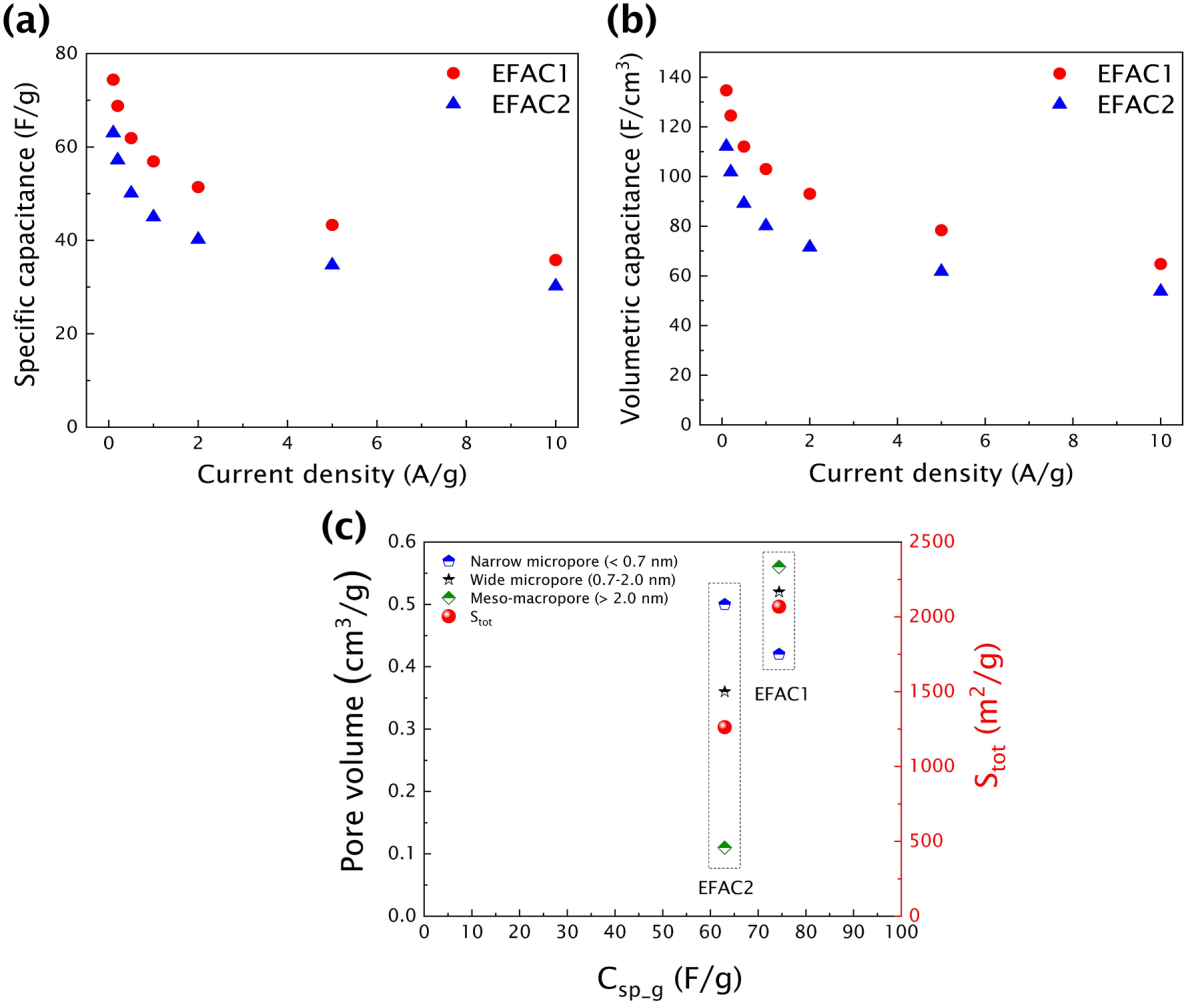
**(a)** Gravimetric capacitance (C_sp_g_), and **(b)** volumetric capacitance (C_sp_v_) of EFAC1 and EFAC2 recorded at 0.1–1.0 A/g, and **(c)** correlation of specific capacitance to the S_tot_ and pore volume of porous CF electrodes.

Specific capacitances (C_sp_g_ and C_sp_v_) of the CF-based electrodes are calculated from galvanostatic charge–discharge profiles and the calculated values are shown in Fig. 5a and b, which show similar tendencies of the change in capacitance with the current density. The measured specific capacitances correlate with the specific surface areas of the CFs. Consequently, the material with the highest S_tot_ shows the highest C_sp_g_ of 74.4 F/g, while the material with the lowest S_tot_ shows the lowest C_sp_g_ of 63.0 F/g at a current density of 0.1 A/g. Moreover, the CF electrodes have an excellent volumetric capacitance of 134.7 F/cm^3^ (EFAC1) and 112.1 F/cm^3^ (EFAC2) at a current density of 0.1 A/g and retain the volumetric capacitances of 64.8 F/cm^3^ and 53.7 F/cm^3^, respectively, even at a high current density of 10 A/g. Porous carbon with large specific surface areas is widely employed for EDLCs to improve gravimetric energy storage capacity; however, high surface area carbon-based electrodes suffer from poor volumetric capacitance due to the low packing density and large void fraction of porous materials ^50,51^. Electrodes fabricated from the as-prepared CFs contain dense carbon spherical particles, having the advantage of high packing density compared to other forms of amorphous carbon ^41^. The CFs that contain carbon spheres provide a compact structure while retaining high porosity that results in high gravimetric and volumetric capacitances.

High specific capacitance and a very small IR drop observed at the beginning of the discharge curves indicate that both CF-based electrodes provide an excellent diffusion path for easier movement of ions between the electrodes and electrolyte during the charge–discharge process and high electrical conductivity showing good performance. However, EFAC1 shows better specific capacitances than EFAC2 as can be seen in the larger area enclosed by the CV curves as well as longer discharge time in GCD, as it has a higher S_tot_ due to the hierarchical porous structure with wider micropores, meso- and macropores. As the EFAC2 has a lower S_tot_ due to narrow micropores and less hierarchical structure, it exhibited relatively a lower specific capacitance.

The correlation between the specific capacitance, S_tot_ and the pore volume of porous CF electrodes is given in Fig. 5c. Even if the S_tot_ of both the materials has a large variance, the difference in their capacitances is minimal. The two different pathways of activation resulted in the difference in properties such as specific surface area, pore structure and surface functionalities. The hierarchical pore structure of EFAC1 aided in achieving higher capacitance due to its larger S_tot_, however, the larger IR drop showed that the rate of diffusion is slower. In EFAC2, though it possesses lower S_tot_ and narrow micropores, the higher -OH functional group on the carbon framework played a crucial role in achieving good capacitance and lower ohmic resistance predominantly through reversible adsorption–desorption mechanism due to its superior interface affinity properties ^52^.

**Figure 6:**
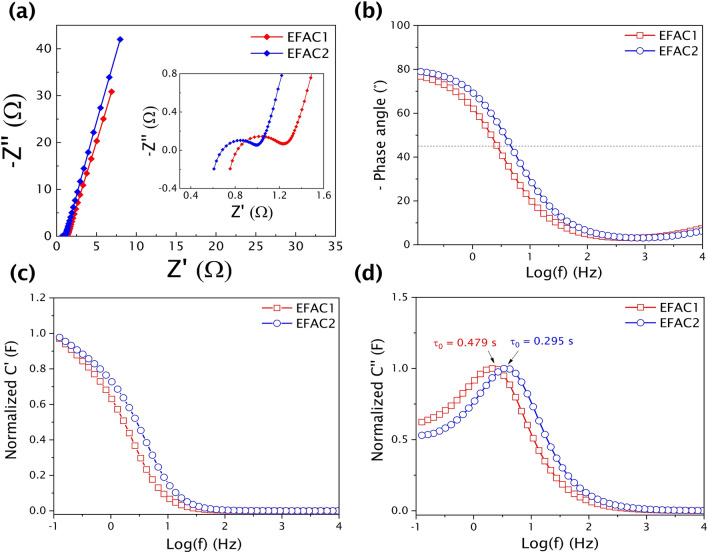
**(a)** Nyquist plots of CF electrode-based supercapacitors (The inset figure shows that the semicircle in the high-frequency range indicates the charge transfer resistance), **(b)** Bode plot of phase angle with frequency, **(c)** Frequency-dependent real capacitance plot (C' Vs frequency), and **(d)** Frequency-dependent imaginary capacitance plot (C" Vs frequency).

The electrochemical behaviour of the two materials was further investigated with the EIS over the frequency range of 100 kHz to 0.1 Hz. The sufficient ion diffusion is confirmed by the Nyquist plot (Fig. 6a). The Nyquist plots show a semicircle in the high-frequency region and a linear shape in the low-frequency region. The insets in Fig. 6a show the high-frequency region zoomed in for visibility. The high-frequency region depicts Faradaic charge transfer resistance on the surface of the porous electrode and more linearity in the low-frequency region indicates that the diffusion-controlled electrode kinetics and this resistance can be modelled as Warburg impedance^53^. The diameter of the semicircle region of the EFAC2 plot is smaller than that of the EFAC1, indicating lower charge transfer resistance. The values of charge transfer resistance obtained from impedance plots are 0.32 Ω and 0.23 Ω for EFAC1 and EFAC2, respectively. Meanwhile, the ability of fast ion diffusion can be judged by the projection of the Warburg type-line on the real axis. In comparison, the EFAC2 plot has a larger Warburg angle (> 45 °) indicating fast electrolyte ion diffusion into the electrodes. The more vertical the straight line at the low-frequency region, the more intensely the supercapacitor behaves like an ideal capacitor. The ESR can be determined from the offsets on the x-axis in the high-frequency region. The ESR obtained from the impedance plots are 1.32 Ω and 1.05 Ω for EFAC1 and EFAC2, respectively. The presence of more hydroxyl functional groups relatively improved the wettability of EFAC2 in the aqueous electrolyte, facilitating the easier diffusion of ions into the carbon electrodes, thus achieving lower charge transfer resistance. The hierarchical porous structure of EFAC1 does not necessarily enhance ionic transport in the CFs and similar behaviour of high capacitance and high ESR was observed and the mechanism of diffusion of electrolyte ions into the HPC was reported ^54^. Therefore, apart from hierarchical porous structure, the type and concentration of surface functional groups play a major role in determining the rate of diffusion of electrolyte ions into the carbon electrodes. This process is crucial in determining the power density of a supercapacitor. Using the ESR value, the maximum power of the device was calculated to be 39.29 kW/kg (EFAC1) and 48.89 kW/kg (EFAC2), respectively.

Figure 6b shows the Bode phase plot (phase angle Vs frequency) of EFAC1 and EFAC2. At high frequencies, the phase angle is almost zero. Below 100 Hz, the phase angle increases rapidly towards a more negative value with decreasing frequency. At the low-frequency region, a phase shift of −76.77° and −79.26° is observed for EFAC1 and EFAC2, respectively, which is close to − 90°, exhibiting nearly ideal capacitive behaviour^55^. The capacitor response frequency corresponds to the phase angle of − 45°, at which the SC transform from purely resistive to purely capacitive behaviour^56^. EFAC2 reached − 45° at a relatively higher frequency than EFAC1, thus revealing the fastest diffusion of ions in EFAC2. The plots of frequency-dependent C' and C" components of the capacitance of both materials are shown in Fig. 6c and d. From the real part of the capacitance (C') Vs frequency plot, we observe that at high frequencies the capacitive behaviour vanishes as the electrolyte ions can have access only to the surface of carbon electrodes. However, at low frequencies, the polarization is slower resulting in the maximum capacitance as the ions can reach the whole surface of the electrodes. In the transition region, the capacitance is limited by the diffusion of ions inside the carbon particle^54^. As discussed before, the EFAC2 exhibits the transition at the highest frequency, therefore the fastest diffusion rate. The peak in the plot of the frequency-dependent imaginary part of capacitance (C") displays a maximum capacitance at a frequency f_0_ which corresponds to the relaxation time τ_0_ (= 1/f_0_). The relaxation time is a measure of how fast the device can be charged/discharged and implies the minimum time needed to discharge all the energy from the device with an efficiency greater than 50% ^57^. Hence the shorter the time, the less limited the charge transport. The relaxation time of EFAC1 and EFAC2 is found to be 479 ms and 295 ms, respectively. The shorter relaxation time of EFAC2 indicates the faster charge/discharge reversibility and efficiency.

**Figure 7:**
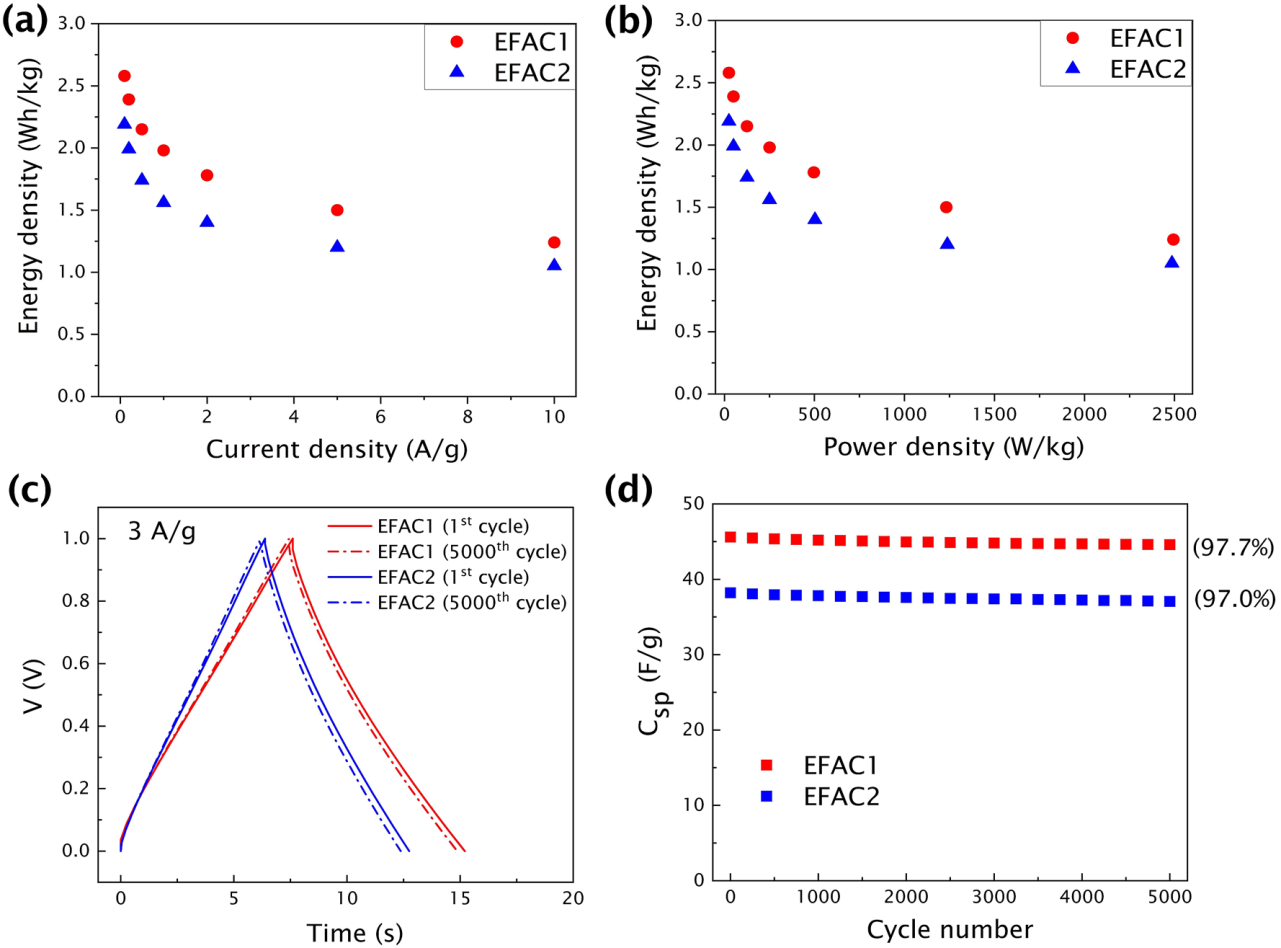
**(a)** Energy densities calculated at different current densities, **(b)** Ragone plot for EFAC1 and EFAC2, **(c)** Charge–discharge profiles of EFAC1 and EFAC2 at 1^st^ and 5000^th^ cycle at a current density of 3 A/g, and **(d)** The cycle life of the supercapacitor measured at a current density of 3 A/g.

The energy densities of supercapacitors are calculated using the specific capacitance obtained from charge–discharge profiles and the results are shown in Fig. 7a. The energy densities of the supercapacitors are 2.58 Wh/kg and 2.19 Wh/kg for EFAC1 and EFAC2, respectively, at the current density of 0.1 A/g. The Ragone plot for the CF-based EDLC is shown in Fig. 7b.

The cyclic stability of the CF-based cells is explored in a 6 M KOH aqueous solution at 3 A/g. The specific capacitances of EFAC1 and EFAC2 at 3 A/g are calculated as 45.6 F/g and 38.2 F/g, respectively and they have slightly decreased to 44.6 F/g and 37.1 F/g, respectively, even after 5000 cycles. The charge–discharge profile remains symmetric after multiple cycles (Fig. 7c), indicating relatively high capacitance retention of 97.7% (EFAC1) and 97.0% (EFAC2) as shown in Fig. 7d. The Coulombic efficiency (η) of the cells is close to 100% for the 5000 charge–discharge cycles at 3 A/g. The exceptional rate capability is potentially due to the porous structure and also the presence of oxygen functional groups that facilitates ion diffusion and the high electrical conductivity that enables fast charge transfer at interfaces and into the pores of the electrodes.

Table 3 presents a literature comparison of the current work with the capacitive performance of porous carbon electrodes produced from various plastic and biomass wastes. The specific capacitances and the capacitance retentions of electrodes (tested in two-electrode configuration) fabricated from waste polyurethane elastomer are comparable to the electrodes produced from other plastic and biomass wastes already reported.

**Table 3:** Literature overview of the capacitive performance of porous carbon electrodes produced from various plastic and biomass wastes.

Precursor	Electrode^1^/ Electrolyte	Specific capacitance, C_sp-g_ (F/g)	No. of cycles	Capacitance retention (%)	Ref
PU elastomer waste (EFAC1; EFAC2)	2E/6 M KOH	74.4; 63.0 (at 0.1 A/g)	5000	97.7; 97.0 (at 3 A/g)	Current work
Polyethyelene terephthalate (PET) waste	3E/6 M KOH	325 (at 0.5 A/g)	5000	91.86 (at 5 A/g)	^30^
Polyethylene (PE) waste	2E/6 M KOH	110 (at 0.05 A/g)	10,000	97.1 (at 2 A/g)	^32^
Polypropylene (PP) waste	3E/6 M KOH	349 (at 0.5 A/g)	10,000	99.0 (at 5 A/g)	^34^
Polystyrene (PS) waste	2E/6 M KOH	69.3 (at 0.5 A/g)	5000	93.6 (at 10 A/g)	^35^
Poly(vinyl chloride) (PVC) waste	3E/6 M KOH	399 (at 1.0 A/g)	1000	96.0 (at 5 A/g)	^58^
Polytetrafluoroethene (PTFE) waste	3E/6 M KOH	313.7 (at 0.5 A/g)	5000	93.1 (at 20 A/g)	^59^
Silica sphere nano array & triblock copolymer P123 template	2E/6 M KOH	54.2 (at 1.0 A/g)	10,000	91.0 (at 0.5 A/g)	^60^
Wheat straw cellulosic foam	3E/6 M KOH	226.2 (at 0.5 A/g)	5000	78.4 (at 0.5 A/g)	^61^
Oxidized lignin	2E/6 M KOH	274.02 (at 0.1 A/g)	5000	88.46 (at 5 A/g)	^62^

^1^ 2E/3E represents two-electrode/ three-electrode electrochemical testing configuration. The three-electrode system typically shows 2–3 times higher capacitances than the two-electrode configuration ^63^.

## Conclusion

Supercapacitor electrodes fabricated from waste PU elastomer-based porous carbon foams exhibit a specific capacitance of 74.4 F/g at a current density of 0.1 A/g. The as-prepared CFs contain spherical carbon particles and have the advantage of high packing density compared to other porous carbon. Hence, CF electrodes display an excellent volumetric capacitance of 134.7 F/cm^3^ at a current density of 0.1 A/g and retain the volumetric capacitance of 64.8 F/cm^3^ even at the high current density of 10 A/g. Low equivalent series resistance and charge transfer resistance obtained from impedance measurements support the ability of the supercapacitor to attain high power. The electrochemical performance of the CFs is not only controlled by the hierarchical pore structure and surface area but also the type and concentration of the oxygen-containing surface functional groups. Both CFs-based EDLCs exhibit Coulombic efficiency near 100% and stable cyclic performance for at least 5000 charge–discharge cycles with good capacitance retention of approximately 97.0% at 3 A/g. These results suggest that the CFs produced from waste polyurethane elastomer templates have potential as EDLCs.

## Supplementary Information

Supplementary Information.

## Data Availability

All data generated or analysed during this study are included in this article. The raw data are available from the corresponding author upon reasonable request.
