# Supplementary material for: Hierarchical porous carbon foam electrodes fabricated from waste polyurethane elastomer template for electric double-layer capacitors
Source: Sci Rep. 2022 Jul 11;12:11786. doi: 10.1038/s41598-022-16006-8 (PMC9276828; doi:10.1038/s41598-022-16006-8)
Supplement: Supplementary file 1 — Supplementary Information. [file 41598_2022_16006_MOESM1_ESM.pdf]

Hierarchical porous carbon foam electrodes fabricated from waste polyurethane elastomer template for electric double-layer capacitors

Mahitha Udayakumar[1,2], Pál Tóth[3], Henrik Wiinikka[4,5], Jaskaran Singh Malhotra[4,6], Blaz Likozar[7], Saso Gyergyek[7], Anett Katalin Leskó[8], Ravikumar Thangaraj[1,2], Zoltán Németh[1]*

[1]Advanced Materials and Intelligent Technologies Higher Education and Industrial Cooperation Centre, University of Miskolc, H-3515 Miskolc, Hungary

[2]Institute of Chemistry, University of Miskolc, H-3515 Miskolc, Hungary

[3]Institute of Physical Metallurgy, Metal Forming and Nanotechnology, University of Miskolc, H-3515 Miskolc, Hungary

[4]RISE Energy Technology Center, Box 726, SE 941 28, Piteå, Sweden

[5]Division of Energy Science, Department of Engineering Sciences and Mathematics, Luleå University of Technology, SE-97187, Luleå, Sweden

[6]DTU Offshore, Technical University of Denmark, Elektrovej, Building 375, 2800 Kongens Lyngby, Denmark

[7]Department of Catalysis and Chemical Reaction Engineering, National Institute of Chemistry, Hajdrihova 19, 1001, Ljubljana, Slovenia

[8]Institute of Energy and Quality Affairs, University of Miskolc, H-3515 Miskolc, Hungary

* Correspondence: kemnemet@uni-miskolc.hu

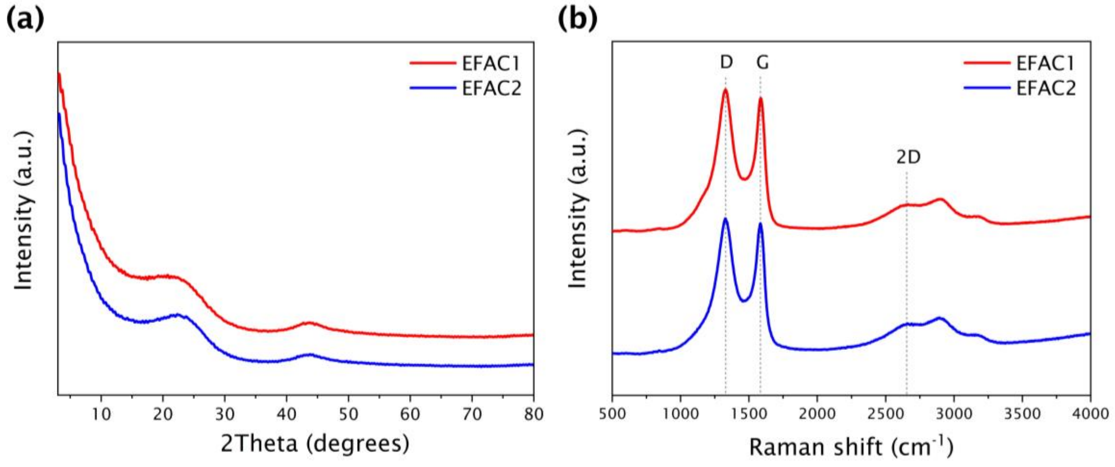

Figure S1. (a) Powder XRD and **(b)** Raman spectra of the CFs.

The structural properties of the CFs were investigated by XRD and Raman analysis. The

XRD diffractogram of the CFs is shown in Fig. S1(a). Both materials exhibited two broadened diffraction peaks around 23° and 44°, which represents the respective (002) and (100) diffraction of the amorphous or turbostratic carbon [1,2]. Figure S1(b) shows the Raman spectra of the CFs. The first-order region in the Raman spectra exhibited two main peaks around 1340 cm^{-1} and 1580 cm^{-1}, corresponding to the D-band (defect-induced band) and G-band (graphitic band) of the carbon material, respectively. The peak position and the intensities of D- and G-bands again confirmed the turbostratic nature of the CFs [2].

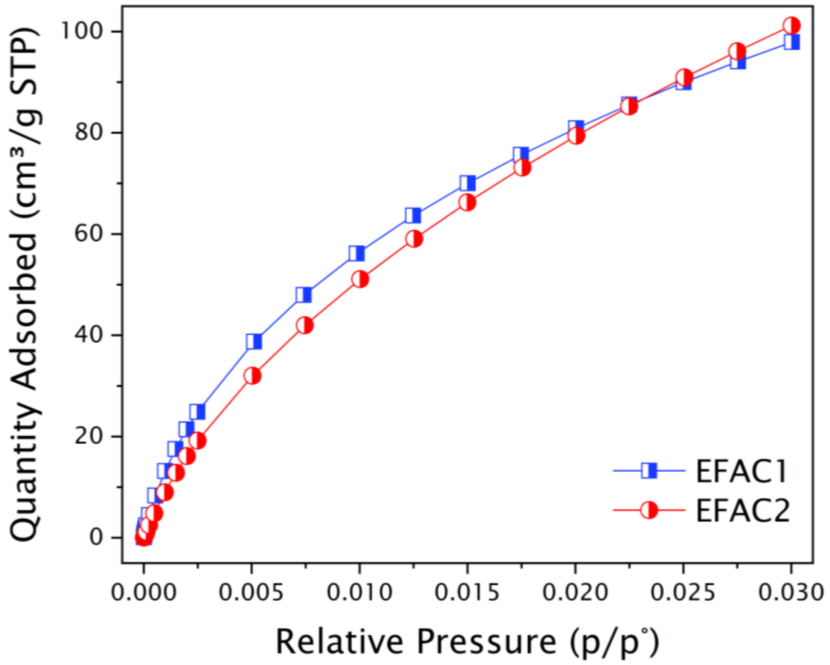

Figure S2. CO_2 adsorption isotherm of carbon foams (CFs) at 273 K.

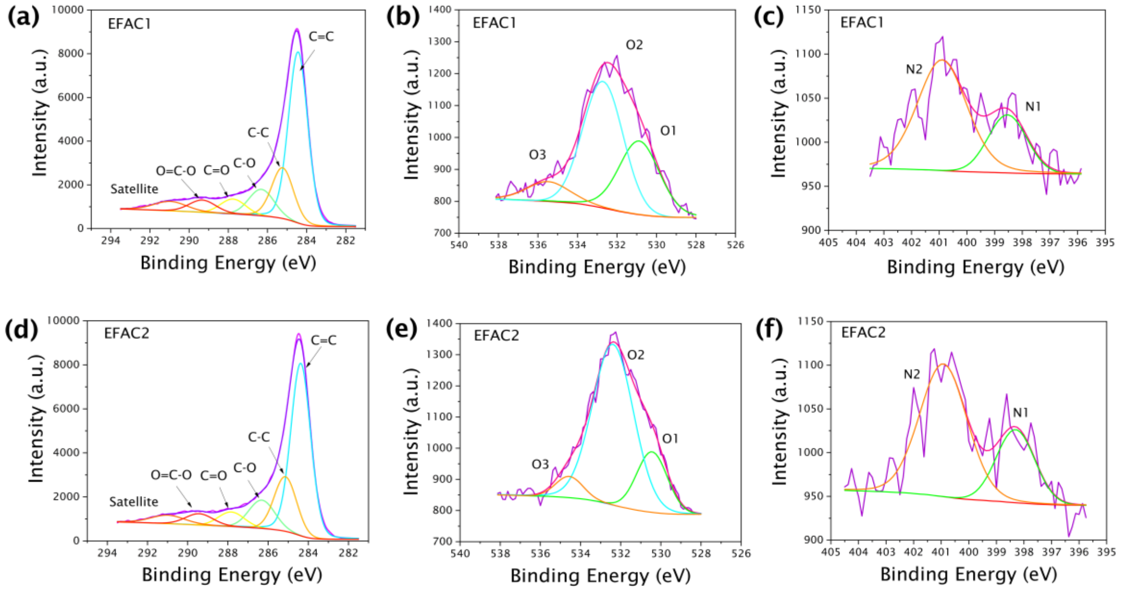

Figure S3. Deconvoluted HR XPS spectrum C 1s **(a, d)**, O 1s **(b, e)** and N 1s **(c, f)** of the CFs.

The deconvoluted XPS spectra of the carbon foams are shown in Fig. S3. The C 1s spectrum of EFAC1 and EFAC2 is decomposed into 6 peaks as shown in Figs. S3(a) and (d). The deconvolution of the C 1s peaks shows the presence of graphitic carbon at 284.4 eV and 285.3 eV in particular with C=C and C-C/C-H bonds, respectively. The graphitic carbon concentration is about 55% in both samples. In addition to graphitic peaks, smaller peaks at higher binding energy are recognized and associated with oxidized carbon atoms, such as a peak at 286.5 eV relate to C-OH bonds, a peak at 287.9 eV relate to C=O/O-C-O bonds, a peak at 289.3 eV relate to O=C-O (COOH/COOR) bonds [3]. The peak at approximately 291 eV is assigned to the satellite peak due to π-π* interactions in benzene-type rings.

The deconvolution results of the O 1s peaks show three major functional groups as presented in Figs. S3(b) and (e). The peak at the binding energy of 530.8 eV is assigned to the quinone-type carbonyl groups (O=C/O-C-O bonds). The peak at 532.7 eV corresponds to the C-OH bonds and the peak at 535.4 eV is related to C-O-C bonds [4]. Based on the peaks at 286.5 eV and 532.7 eV, it seems the relative concentration of the hydroxyl functional group of EFAC2 is higher than EFAC1, which enhanced the wettability of EFAC2.

XPS spectra of nitrogen N 1s (Figs. S3(c) and (f)) were measured for both the samples with similar intensities reflecting the surface concentration of 1-2 at.%. These spectra are decomposed into two peaks. The peak at 398.5 eV is smaller (23 % of the total intensity of N 1s spectra) and it corresponds to pyridinic N. The peak at 400.9 eV is larger (77 % of the total intensity of N 1s spectra) and it is related to graphitic N [5].

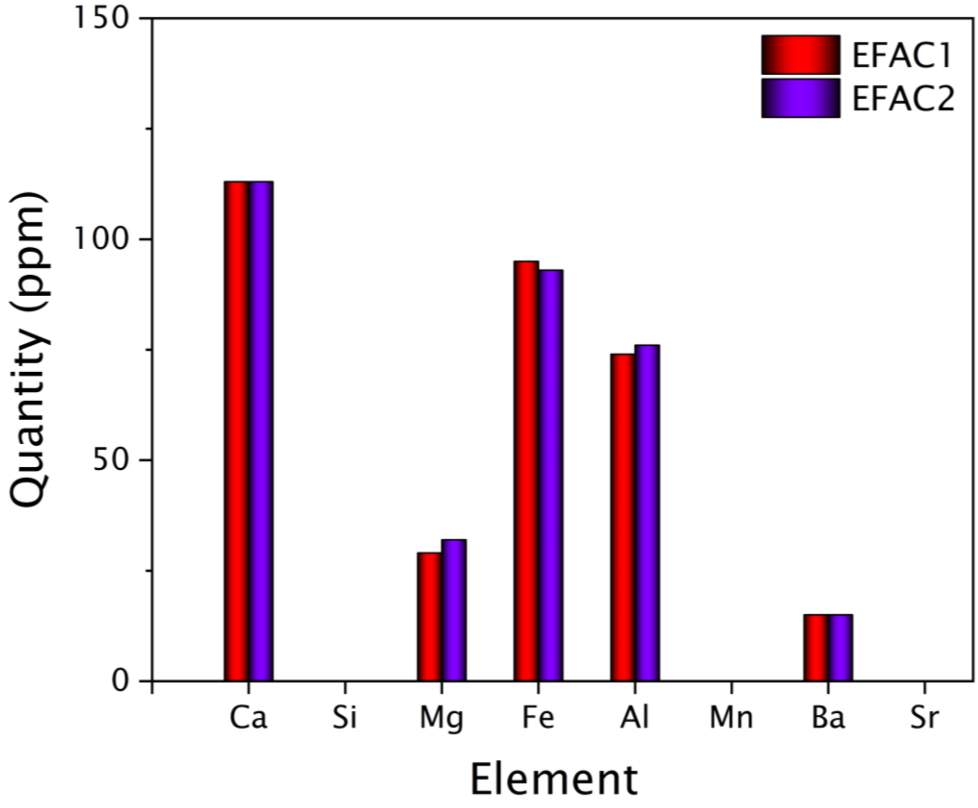

Figure S4. Metallic impurities (in ppm) of CFs by ICP-OES.

References

1. Nam, G., Choi, S., Byun, H., Rhym, Y. M. & Shim, S. E. Preparation of macroporous carbon foams using a polyurethane foam template replica method without curing step. *Macromol. Res. 2013 219* **21**, 958–964 (2013).
2. Udayakumar, M. *et al.* Synthesis of activated carbon foams with high specific surface area using polyurethane elastomer templates for effective removal of methylene blue. *Arab. J. Chem.* **14**, 103214 (2021).
3. Jovanovic, Z. *et al.* The role of surface chemistry in the charge storage properties of graphene oxide. *Electrochim. Acta* **258**, 1228–1243 (2017).
4. Umezawa, S. *et al.* Supercapacitor electrode with high charge density based on boron-doped porous carbon derived from covalent organic frameworks. *Carbon N. Y.* **184**, 418–425 (2021).
5. Scardamaglia, M. *et al.* Spectroscopic observation of oxygen dissociation on nitrogen-doped graphene. *Sci. Rep.* **7**, 1–11 (2017).
